# Molecular analysis of *CFTR* gene mutations among Iraqi cystic fibrosis patients

**DOI:** 10.1186/s43042-021-00164-x

**Published:** 2021-05-11

**Authors:** Asal Gailan Abdul-Qadir, Bassam Musa Al-Musawi, Rabab Farhan Thejeal, Saad Abdul-Baqi Al-Omar

**Affiliations:** 1 Department of Medical Genetics, Kamal Alsamaraey Hospital (Infertility Center), Ministry of Health, Baghdad, Iraq; 2 Department of Pathology and Forensic Medicine, College of Medicine, University of Baghdad, Baghdad, Iraq; 3 Children Welfare Hospital, Medical City, Baghdad, Iraq; 4 Department of Pediatrics, College of Medicine, University of Baghdad, Baghdad, Iraq; 5 Department of Pathology and Forensic Medicine, College of Medicine, University of Basra, Basra, Iraq

**Keywords:** Cystic fibrosis, *CFTR*, Mutations, 3120+1G>a, W1282X, F508del, Iraq

## Abstract

**Background:**

Cystic fibrosis (CF) is an autosomal recessive multisystem disease that results from mutation(s) of the cystic fibrosis transmembrane conductance regulator (*CFTR*) gene. More than 2100 mutations and polymorphisms have been reported in this gene so far. Incidence and genotyping of CF are under-identified in Iraq. This study aims to determine the types and frequencies of certain *CFTR* mutations among a sample of Iraqi CF patients. Two groups of patients were included: 31 clinically confirmed CF patients in addition to 47 clinically suspected patients of CF. All confirmed patients had typical, moderate-severe clinical presentation and course of the disease. Molecular analysis was performed on the majority of enrolled patients using the CF-stripAssay® kit supplied by ViennaLab diagnostics, GmbH, Austria.

**Results:**

The mutation-detection rate from the tested 34 mutations in this study was 19.5% and the 8 detected mutations were as follows: 3120+1G>A and W1282X were found in 3 (4.17%) patients each; F508del and R1162X were found in 2 (2.78%) patients each; 3272-26A>G, R347P, I507del, and 2183AA>G were found in 1 (1.38%) patient each. Polymorphic variants of IVS8, namely 5T, 7T, and 9T, were detected in ~ 70%. These results were nearly similar to what was reported in regional countries.

**Conclusion:**

Cystic fibrosis seems to be not rare as previously thought. 3120+1G>A and W1282X are the two most commonly detected mutations. F508del needs to be included in all future tests, while the I507del mutation was uniquely reported in this study but not in regional studies.

## Background

Cystic fibrosis (CF; MIM #219700) is an inherited autosomal recessive disorder with multisystem manifestations [1]. It is estimated to affect as many as 1 in 2000–3000 Caucasian newborns in Europe and USA [2] and 1 in 2560 to 1 in 15,876 in the Middle East. The incidence differs concerning the ethnic and geographic background as well as the degree of consanguinity [3].

CF is caused by mutations of the cystic fibrosis transmembrane conductance regulator (*CFTR*) gene that spans about 250 kb of genomic DNA; it is located at chromosomal region 7q31.2 and contains 27 exons. More than 2100 *CFTR* mutations and polymorphisms have been detected to date, with variable frequencies [4].

Generally, mutations associated with clinical manifestations are grouped into six main classes depending on how they affect protein structure and function. Mutation class I–III are considered severe mutations, while class IV–VI mutations are considered less severe ones [5].

The major manifestations of the disease involve mainly the respiratory and gastrointestinal (GI) systems (chronic productive cough, dyspnea, recurrent lower respiratory tract infections, pancreatic insufficiency, malabsorption, fatty diarrhea, and distal intestinal obstruction syndrome). In children, meconium ileus and failure to thrive are additional manifestations. Other features can also be present like kidney and endocrine gland involvements in addition to infertility in both males and females [6].

Diagnosis depends on the patient’s suggestive clinical presentation, or positive newborn screening test (elevated immunoreactive trypsinogen level in blood) plus laboratory evidence of CFTR dysfunction like two positive sweat chloride tests (> 60 mmol/L of chloride in sweat) obtained on separate days or identifications of two *CFTR* mutations or abnormal nasal potential difference [1].

*CFTR* molecular genetic testing is important for the diagnosis especially for inconclusive clinical/biochemical results and in prenatal diagnosis. Direct gene analysis relies on either testing for the presence or absence of known mutations, which can be achieved by a panel test, or scanning the samples for any deviation from the wild sequence (for unknown mutations) using sequencing methods [7].

CF has largely been under-diagnosed in Iraq due to a low index of suspicion and inadequate facilities for diagnosis in some regions. To the best of our knowledge, no published data are available on the incidence of CF in Iraq.

This investigation aims to detect the types and frequencies of certain *CFTR* mutations among a group of clinically diagnosed Iraqi cystic fibrosis children.

## Methods

In this cross-sectional study, two groups of Iraqi Arab CF patients were recruited. The first group included clinically confirmed patients of CF [suggestive clinical features and biochemical abnormalities, plus positive sweat chloride test (> 60 mmol/L)]. They were recruited mostly from the Gastroenterology and Liver Diseases Outpatient Clinic, Children Welfare Hospital, Baghdad, Iraq. They attended this tertiary center from all over the country, but mostly from Baghdad. Molecular analysis was performed on the majority of them using CF-stripAssay® kit supplied by ViennaLab diagnostics, GmbH, Austria. The assay covers 34 mutations and 3 polymorphic variants in the *CFTR* gene allocated on 2 strips. These mutations are CFTRdel2, 3 (21 kb), I507del, F508del, 1717-1G>A, G542X, G551D, R553X, R560T, 2143delT, 2183AA>G, 2184delA, 2184insA, 2789+5G>A, R1162X, 3659delC, 3905insT, W1282X, N1303K, G85E, 394delTT, R117H, Y122X, 621+1G>T, 711+1G>T, 1078delT, R334W, R347H, R347P, A455E, 1898+1G>A, 3120+1G>A, 3272-26A>G, Y1092X, 3849+10kbC>T, in addition to 3 polymorphisms, namely IVS8 5T, 7T, and 9T “1201-12T[n] (*n* = 5/7/9)”.

The second group included clinically suspected patients of CF; all reside in Basra City in the South of Iraq and were referred to a genetic laboratory for genetic testing using the same kit. Their clinical features and biochemical findings were not available; besides, the sweat chloride test was not available in that city and thus was not performed. They were included in this study for the sake of collecting all available molecular data of CF in Iraqi patients. The total number of enrolled patients was, therefore, 72 from the two groups.

Molecular diagnosis included DNA extraction from peripheral blood samples, multiplex PCR amplification using biotinylated specific primers, followed by hybridization of amplification products to test strips containing allele-specific oligonucleotide probes immobilized as an array of parallel lines. The bound biotinylated sequences are detected using streptavidin-alkaline phosphatase and color substrates.

Verbal consents were taken from the patients’ parents and the study was approved by the Research Ethics Committee, Dept. of Pathology and Forensic Medicine, College of Medicine, University of Baghdad (document #1356 on October 23^rd^, 2019) as well as from the Ethical Committee of the Ministry of Health, Baghdad, Iraq (document #45961 in November 25^th^, 2019).

## Results

The current study recruited two groups of patients. The first group included 31 previously diagnosed CF patients; their ages ranged between 3 months and 15 years (mean ± SD = 4.6 ± 4.02) years; 18 (58.06%) of them were males and 13 (41.94%) were females (M:F ratio of 1.38:1). Most [27/31 (87.1%)] patients were products of consanguineous marriages; 5/31 (16.13%) patients had a positive family history of CF.

The majority [16 (51.62%)] of patients presented during the first year. The most frequent clinical presentation was typical (combined pulmonary and GI manifestations) (chronic cough with dyspnea, recurrent lower respiratory tract infection, and greasy stool) as seen in 27 (87.1%) patients (Tables 1 and 2).

**Table 1:** Basic demographic and clinical features of 31 Iraqi CF patients

Variables	Variables	No. (%)
Gender	Male	18 (58.06%)
	Female	13 (41.94%)
Consanguinity	Positive	27 (87.1%)
	Negative	4 (12.9%)
Family history	Positive	5 (16.13%)
	Negative	26 (83.87%)
Residence in Iraq	North	1 (3.2%)
	Middle	26 (83.9%)
	South	4 (12.9)
Clinical picture	Typical manifestations	27 (87.1%)
	Atypical (one system) manifestations	4 (9.68%)
Age at presentation	≤ 1month	14 (45.17%)
	> 1 month–≤ 1 year	16 (51.62%)
	> 1 year	1 (3.23%)

**Table 2:** Clinical findings of 31 Iraqi CF patients

Clinical findings	No. (%)
Chronic cough	28 (90.3%)
Recurrent lower respiratory tract infections	28 (90.3%)
Greasy stool	26 (83.8%)
Palpable liver	16 (51.6%)
Chest wheezes and crepitations	13 (41.9%)
Abdominal distention	5 (16.1%)
Abdominal pain and vomiting	1 (3.2%)
Salty sweat	1 (3.2%)

All patients included in this group had a sweat chloride value ranging between (61 and 132 mmol/L) with a mean ± SD of 97.13 ± 21.51 mmol/L; 18 (58.1%) patients had elevated liver enzymes (ALT, AST, and ALP); 13 (41.9%) had elevated total serum bilirubin, while 12 (38.7%) patients had a low total serum protein. Sputum culture revealed *Pseudomonas aeruginosa* in 6 (19.3%) patients and *Klebsiella* spp. in 2 (6.5%) patients; 26 (83.9%) patients had steatorrhea (Table 3).

**Table 3:** Basic investigations results of 31 Iraqi CF patients

Lab. Test		No. (%)
Sweat chloride test	Range = 61–132 mmol/L Mean = 97.13 mmol/L	31 (100%)
Abnormal liver function tests	Elevated ALT, AST, ALP	18 (58.1%)
	Increased total serum bilirubin	13 (41.9%)
	Low total serum protein	12 (38.7%)
Sputum culture	*Pseudomonas aeruginosa*	6 (19.3%)
	*Klebsiella spp.*	2 (6.5%)
	No growth	15 (48.4%)
	Not performed	8 (25.8%)
Stool test for presence of fat	Positive (5+ fat droplet)	26 (83.9%)
	Negative (no fat)	5 (16.1%)

*ALT* alanine aminotransferase, *AST* aspartate aminotransferase, *ALP* alkaline phosphatase

Complications encountered throughout the course of the disease are summarized in Table 4.

**Table 4:** Frequency of complications among 31 studied CF patients

Complications	No.	%
Pneumonia	27	87.1%
Growth failure	20	64.5%
Hypertrophic osteoarthropathy (finger clubbing)	17	54.8%
Meconium ileus	12	38.7%
Bronchiectasis	5	16.1%
Death	4	12.9%
Distal Intestinal obstruction syndrome	3	9.7%
Hepatic steatosis	1	3.2%
Chronic pancreatitis	1	3.2%
Nasal polyp(s)	1	3.2%
Aspergillosis	1	3.2%

Molecular analysis was performed on 25 CF patients in this group; the remaining 6 patients either died before blood aspiration or could not attend due to COVID-19 lockdown. Results revealed the presence of 3 mutations in 5/25 (20%) patients and the presence of IVS8 polymorphic variants (5T, 7T, 9T) in 15/25 (60%) patients while 5/25 (20%) patients did not show any finding.

The detected mutations in this group included F508del mutation (exon 10) and W1282X mutation (exon 20) and they were found in 2 (8%) patients each in addition to R1162X mutation (exon 19), which was found in 1 (4%) patient only. All detected mutations 5/5 (100%) were in the homozygous state and they all presented with typical combined respiratory and GI manifestations during the first year of life; 4/5 (80%) of them were products of consanguineous marriages, 1/5 (20%) had a positive *P*. *aeruginosa* sputum culture, and all 5/5 (100%) patients suffered from failure to thrive.

The second group of patients enrolled in this study included 47 clinically suspected patients of CF, all residing in Basra City in the very south of Iraq; their ages ranged between (1 month and 15 years) years with a mean ± SD of 2.74 ± 3.19 years. Of them, 30 (63.8%) patients were males and 17 (37.2%) were females with a M:F ratio of 1.76:1. The overall M:F ratio for both groups in this study was 1.6:1 [48 (66.7%) males and 30 (33.3%) females].

Molecular analysis of these patients showed that 9 (19.15%) patients had 7 detectable mutations, all in homozygous states as follows: 3120+1G>A in 3/47 (6.38%) patients, while I507del, W1282X, 2183AA>G, R1162X, 3272-26A>G, and R347P were detected in 1 (2.13%) patient each. IVS8 polymorphic variants were also detected as follows: 7T/7T in 25/47 (53.2%) patients, 7T/9T in 8/47 (17%) patients, 9T/9T in 4/47 (8.5%) patients, while 5T/7T was seen in 1/47 (2.13%) patient only.

Table 5 shows molecular data of both groups including all 72 molecularly tested Iraqi CF patients.

**Table 5:** *CFTR* gene mutations/variant detected in 72 molecularly tested CF Iraqi patients

Genotype	Mutation Class	Position	Nucleotide variation	Protein change	Zygosity	No.(%) of cases
Mutations						
3120+1G>A^a^	Class I	Intron 16	c.2988+1G>A	–	homozygous	3(4.17%)
W1282X^b^	Class I	Exon 20	c.3846G>A	p.Trp1282X	Homozygous	3(4.17%)
F508del^c^	Class II	Exon 10	c.1521_1523delCTT	p.Phe508del	Homozygous	2 (2.78%)
R1162X^b^	Class IV	Exon 19	c.3484C>T	p.Arg1162X	Homozygous	2(2.78%)
3272-26A>G	Class V	Intron 17	c.3140-26A>G	–	Homozygous	1(1.38%)
R347P	Class IV	Exon 7	c.1040G>C	p.Arg347Pro	Homozygous	1(1.38%)
I507del	Class II	Exon10	c.1519_1521delATC	p.Ile507del	Homozygous	1(1.38%)
2183AA>G	Class I	Exon 13	c.20512052delAAinsG	p.Lys684SerfsX38	Homozygous	1(1.38%)
Total cases with detected mutations						**14**/**72** (**19**.**44**%)
c.1210-12T[n] background						
IVS8 7T/7T	–	Intron 8	c.1210-12 T[7]	–	Homozygous	36 (50%)
IVS8 7T/9T	–	Intron 8	c.1210-12 T[7] /[9]	–	Heterozygous	10 (13.9%)
IVS8 9T/9T	–	Intron 8	c.1210-12 T[9]	–	Homozygous	5 (6.94%)
IVS8 5T/7T	–	Intron 8	c.1210-12 T[5] /[7]	–	Heterozygous	2 (2.78%)
Total cases with detected polymorphic variants						**53**/**72** (**73**.**62**%)
Unidentified						5 (6.94%)

^a^All 3 cases had additional 7T/7T variant

^b^One case had an additional 7T/7T variant

^c^2 cases had additional 9T/9T variant

## Discussion

The diagnosis of CF in Iraq is challenging due to physicians’ low index of suspension. This disease was thought to be rare in this region and is similar in its presentation to other more common diseases in Iraq like tuberculosis, celiac disease, and immunodeficiency states. Lack of CF neonatal screening program and non-uniform availability of sweat chloride test or genotyping makes confirmation of clinical suspicions rely on clinical response to some available therapies like pancreatic enzyme supplementation, long-term course of the disease, and diagnosis by exclusion.

Interest in this disease has grown in the last decade as more patients are being discovered in Iraq, mostly in pediatric age groups. However, no published data or formal figures are available on the incidence and impact of CF in Iraq.

One unpublished study has been performed by Abbadi AF and Al-Janabi MK in 2016 that focused mainly on clinical characteristics and course of the disease of forty CF Iraqi children. A recently published study was conducted on 30 CF Iraqi children and analyzed exon 10 and exon 17a of *CFTR* gene using Sanger sequencing without clinical correlation. They found 2 mutations and one polymorphic variant in 17 CF patients, namely F508del in 5 (16.6%) patients, and S466X in 1 (3.3%) patient and M470V polymorphism in 11 (36.6%) patients [8]; F508del was the only one tested for in the current study.

In this cross-sectional study, we described the demographic data, presentation, investigations, and complications of the clinically confirmed patients with CF. The overall M:F ratio of both groups was 1.6:1, which is nearly similar to that found in Bahrain, Saudi Arabia, Jordan, and Egypt with a M:F ratio of 1.37, 1.33, 1.32, and 1.5 respectively [9]. All patients enrolled in this study were children.

Parental consanguinity among 31 CF patients was 87.1%. This is higher than the average consanguinity rates among Iraqi general populations (40–49%) [10] but similar to the rates reported for other hereditary conditions in Iraq [11]. This finding also helps to explain the increasing occurrence of rare conditions such as CF and familial aggregation of patients which is seen in 5 (16.13%) patients, similar to that reported in other regional countries [12, 13].

Almost all patients 30/31 (96.78%) presented during the first year reflecting disease severity. This was nearly similar to a study from Egypt where 86% of patients presented during the first year of life [14].

Mutational analysis of all tested patients in this study (*n* = 72) showed that 3120+1G>A and W1282X were the most frequently detected mutations as seen in 3 (4.17%) patients each. The former is known as the African CF mutation and has a frequency of 12.2% in African-American people [15] and was reported in an Iranian study with a similar frequency as ours [16]. The other mutation, W1282X, was reported as the most common mutation in patients from Arab ethnic backgrounds [17] and has a similar frequency to an Egyptian study [12]. This result was different from what was detected in a recently published Iraqi study which revealed that F508del mutation was the most commonly encountered mutation in a sample of 30 Iraqi CF patients as seen in 5 (16.6%) patients [8]. This mutation was the second most common mutation detected in the current study along with R1162X with a frequency of 2.78% as detected in 2 patients each.

F508del is considered the most common *CFTR* mutation in other Arab countries like Lebanon (34%) [18] and Egypt (58%) [12], while R1162X has only been reported in Egypt in 3 patients (6%) [12] but was the second-ranking mutation in the previously published Iraqi study, where 5(16.6%) patients had this mutation [8]. For these reasons, this mutation needs to be included in all future tests.

The other 4 mutations detected in this study were seen in one patient only with a frequency of 1.38% each and included (3272-26A>G, R347P, I507del, and 2183AA>G).

3272-26A>G mutation, which creates an alternative acceptor splice site in intron 17a, is considered the second most common mutation after F508del in the Belgian CF population with a frequency of 3.8% [19]. Al-Abadi et al study showed this mutation in one Jordanian patient [20].

Searching published reports from the Arab world showed that R347P mutation was only reported in Egypt in 2% of patients [12] along with the current study. The worldwide frequency of R347P mutation is about 0.2% and it is mostly found in the south of Bulgaria [21].

I507del mutation is only reported in this study but not in other regional countries, while 2183AA>G was detected in Jordan (6%) [20], Iran (7%) [13], Turkey (2.5–4.9%) [22], and the Northeast of Italy (9.3%) [23].

All patients with detected mutations from the first group (*n* = 5) presented during the first year which is the usual age of presentation of CF patients [24]; all had abnormally high levels of sweat chloride values and failure to thrive which may necessitate aggressive management to slow the progress and reduce potential complications. One patient in this study had R1162X mutation and presented with milder pulmonary manifestations. This mutation results in a protein with a residual regulatory region that may be partially functioning in the lung tissues [25].

Over the years, the IVS8 c.1210-12T[5_9] (polypyrimidine tract in intron 8) and adjacent c.1210-35_1210-12GT[8_13] (TG repeat tracts) in the *CFTR* gene have received much more attention due to their potential roles in the development of male infertility. The polymorphic IVS8 c.1210-12T[5_9] consists of three common variants, namely 5T, 7T, and 9T, and this locus functions as the acceptor site of alternative splicing of *CFTR* exon 9 [26].

The presence of some polymorphic variants is not by itself pathogenic, but some variants are associated with the presence of certain splice mutations. The shortest (5T) is associated with the highest rate of incomplete transcripts. mRNA without exon 9 results in immature CFTR proteins with improper function [27].

The 7T allele can be found in normal individuals with a relative frequency of 80% in Caucasians [28] and 90% in East Asians [29]. In the current study, IVS8 7T/7T variant was detected in 50% of patients which was nearly similar to what was reported in a study from Iran (60%); IVS8 7T/9T and 5T/7T heterozygous states were detected in 16.7% in this study but in 30% in the same study from Iran [30]; the same applies to 9T/9T variant, which was detected in 6.94% in the current study but more in the Iranian study (10%) [30]. This particular variant was also encountered in association with F508del mutation in two patients, unlike the Lebanese study that showed 66.7% of the F508del was associated with 7T allele [31].

Some mutations detected in this study were not associated with any of the tested c.1210-12T[5T/7T/9T] variants in the used kit. The presence of mutations without a detected variant is expected as other variants have not been tested, which may suggest that some specific Iraqi combinations may exist and be associated with novel pathogenic variants in the same allele.

Differences in allele frequencies and clinical presentation could be related to the considerable heterogeneity in *CFTR* mutations, a variation of these mutations in different populations, in addition to the interaction of genetic and environmental factors [19].

All detected mutations have a high genotype/phenotype correlation as all patients express typical and moderate-severe clinical presentation and disease course.

Based on these findings, the currently used kit can diagnose about 20% of patients; the remaining 80% require further testing for large rearrangements with quantitative methods. Such methods could include multiplex ligation-dependent probe amplification (MLPA) or next-generation sequencing (NGS) with an adapted algorithm and specific sequencing of well-known deep intronic variants. These tests could be the next step to complete the *CFTR* gene analysis of molecularly undiagnosed patients. They may reveal probable novel mutations as Iraq, especially the central parts, is well known to harbor heterogeneous mutations from all over the world [32] as it was once the center of attention in education and trade.

## Conclusions

CF is not rare in Iraq as previously thought. Diagnosis remains problematic in some patients. A lengthy multistep molecular diagnostic strategy is still recommended till we build a database that enables shorter and less expensive testing. Synthesis of a custom-made panel for the most commonly encountered mutations can be used for *CFTR* mutation detection in this region of the world. More studies on a larger number of CF patients are necessary to determine the incidence and molecular data of CF in Iraq. This can help assist in rapid detection and solve diagnostic hardships as well as it aids in the nearly forgotten prenatal diagnosis.

## Data Availability

Data and material are openly available in a public repository.

## Abbreviations

ALP: Alkaline phosphatase
ALT: Alanine aminotransferase
AST: Aspartate aminotransferase
CF: Cystic fibrosis
CFTR: Cystic fibrosis transmembrane conductance regulator
M:F ratio: Male to female ratio
MIM: Mendelian Inheritance In Man
MLPA: Multiplex ligation-dependent probe amplification
NGS: Next-generation sequencing
